#  A Population-based study of dementia in the oldest old: the Monzino 80-plus Study

**DOI:** 10.1186/1471-2377-11-54

**Published:** 2011-05-25

**Authors:** Ugo Lucca, Mariateresa Garrì, Angela Recchia, Giancarlo Logroscino, Pietro Tiraboschi, Massimo Franceschi, Chiara Bertinotti, Anna Biotti, Elena Gargantini, Marilena Maragna, Alessandro Nobili, Luca Pasina, Carlotta Franchi, Emma Riva, Mauro Tettamanti

**Affiliations:** 1 Laboratory of Geriatric Neuropsychiatry, Department of Neuroscience, Istituto di Ricerche Farmacologiche "Mario Negri", Milano, Italy; 2 Department of Neurological and Psychiatric Sciences, University of Bari, Bari, Italy; 3 Department of Neurology, Ospedale Niguarda Ca' Granda, Milano, Italy; 4 Department of Neurology, IRCCS Multimedica, Castellanza (Varese), Italy; 5 Laboratory of Quality Assessment of Geriatric Therapies and Services, Department of Neuroscience, Istituto di Ricerche Farmacologiche "Mario Negri", Milano, Italy

## Abstract

**Background:**

Despite being the fastest growing and the most cognitively impaired age group, the oldest olds are under-represented in clinical research. The purpose of this study was to describe the design, methods, and baseline characteristics of the survey population and investigate possible differences in demographic, cognitive, functional, and behavioral characteristics between oldest old with and without any performance on cognitive tests and between oldest old alive and those deceased prior to the interview.

**Methods:**

The Monzino 80-plus Study is a prospective door-to-door population-based survey among 80 years or older residents in the municipalities in the province of Varese, Italy. Dementia cases were identified with a one-phase design. Trained psychologists interviewed both the subject and a proxy informant. The interview included a comprehensive standardized questionnaire together with an array of rating scales and a multidomain cognitive battery to assess cognitive and functional ability, behavioral disturbances and mood.

**Results:**

Information was available for 2,139 of the 2,428 registered individuals aged 80 years or older. Main baseline characteristics of the population are reported and discussed. In comparison with those living, elderly persons who had died before the first visit were older, had twice the rate of institutionalization, poorer cognitive performance and competence, and significantly greater instrumental and basic functional disability. The percentage of elderly persons, alive at baseline, without Mini-Mental State Examination rose rather evenly with age. Moreover, they had significantly worse cognitive competence and functional ability, and reported higher prevalences of depressive symptoms and problem behaviors than those with Mini-Mental State Examination.

**Conclusions:**

Prospective investigation of a large population of oldest old can contribute significantly to understanding the relations between age, cognitive decline, and dementia occurrence. Use of informant-based instruments in surveys in the oldest old is crucial in assessing everyday functioning and changes, especially in participants with no cognitive test performance available. Failure to include information on deceased elderly would underestimate, increasingly with age, the prevalence of cognitive and functional disability in the elderly population.

## Background

Cognitive functioning declines with advancing age. Though the large majority of dementia sufferers are among the oldest old [1,2], "patients with dementia that are included in clinical research are systematically younger than patients in the general population" [3]. This over-representation of individuals below age 70 and under-representation of those 80 years or older results in an age gap that may be an important source of bias [3]. In the near future this age gap will become even wider since, with the continuous ageing of the population, individuals 80 years or older are the fastest growing segment of the elderly population [2,4].

Conducting studies in the oldest old is a major problem in epidemiological research and the number of over-eighties investigated in surveys on dementia is usually small [2,5]. Prevalence studies carried out in Italy have shown a large disproportion between the affected and the elderly included: while most of those affected by dementia were 80 years or older, the elderly of the same age group investigated were less than 20 percent, even less than the individuals younger than 60 [2]. Due to these small numbers, prevalence and incidence estimates in the oldest old fluctuate widely and the evidence is often insufficient to reach confident conclusions. In addition, difficulties with the diagnoses of dementia syndrome and type are positively associated with age. Accordingly, the relationship between neuropathological findings and dementia has also been shown to vary with age [6-8].

Accurate estimates of dementia occurrence in this age group are critical for a better understanding of cognitive decline and dementia and for planning medical care. The Monzino 80-Plus Study was established to estimate the prevalence, incidence and progression of dementia and cognitive impairment and to investigate their determinants in the very old (80-plus) in a population-based setting. Even though disease and disability are highly prevalent among the oldest old and form the background against which cognitive decline occurs, information on the health status of this age segment from large cohorts of the elderly population is lacking. Here, we describe the study design, methods, and baseline characteristics of the study population and address important methodological issues in performing epidemiological studies in the very old, such as possible differences in demographic, cognitive, functional, and behavioral characteristics between oldest old with and without any cognitive test performance and between oldest old alive and those deceased at the time of the interview.

## Methods

### Study population and setting

**Figure 1:**
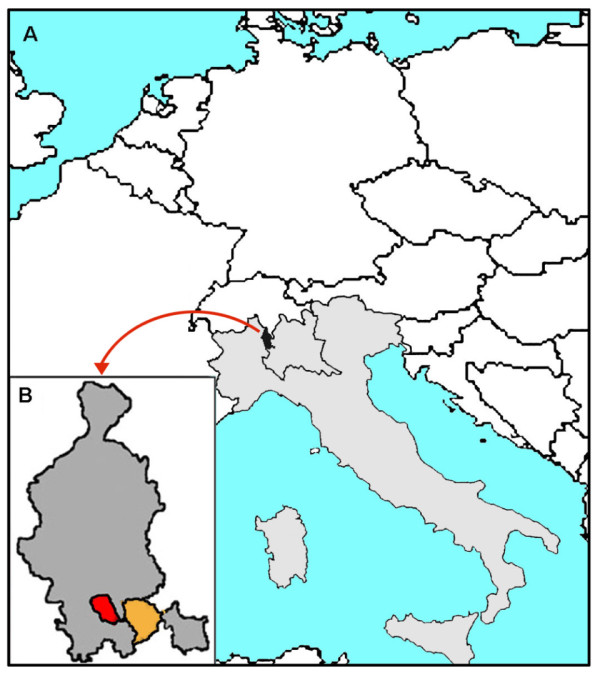
**Map of the study area of the Monzino 80-plus Study:** A. Europe, Italy (in grey) and, within the borders of the Lombardy region, the province of Varese (in black). B. The study area: the province of Varese (in grey) and the area of the eight municipalities initially investigated: in red Gallarate and in orange the seven municipalities of the lower Olona valley (Fagnano Olona, Gorla Maggiore, Solbiate Olona, Gorla Minore, Olgiate Olona, Marnate, and Castellanza)

The Monzino 80-plus Study is an ongoing, prospective door-to-door population-based survey among all residents 80 years or older in eight neighboring municipalities in the province of Varese, Italy (Figure 1). Some 114,000 people reside (2009) in this predominantly urban area, which was traditionally industrial (textile and mechanical industry), though also rural when the present study cohort was of working age. At present employment is mainly in the industrial and service sectors. Migration from other world regions is recent and mostly involves young people, so all but six elderly people included in the study (three French, two German, and one Albanian mother tongue but all speaking Italian fluently) were of Italian origin. The study population was composed mainly of local natives (64.7%) and elderly from the same Lombardy region (8.2%), but there were earlier immigrants from northeastern (14.1%), northwestern (2.3%), central (2.2%), and southern (7.4%) Italy. Because of the age group investigated, the stability of the study population is high and its age structure similar to that of the general Italian elderly population.

### Study design

Lists of residents were obtained from the municipal registry offices where births and deaths are officially recorded. All registered individuals 80 years or older residing in Castellanza, Gorla Maggiore, Gorla Minore, Marnate, Olgiate Olona, and Solbiate Olona and 85 years or older residing in Fagnano Olona on the prevalence day (February 12, 2002) were eligible for the study. To increase the number of very old people and consequently the confidence in the distributions, the survey was subsequently extended to all registered individuals aged 90 or older residing in Gallarate on January 1, 2005 and, recently, to all those aged 100 or older residing in the remaining municipalities of the province of Varese in 2009 (n = 272). In view of the small number of men in the age group 95-99, the study was further extended to include a random sample (n = 110) of men aged 95-99, residents in the same municipalities as the centenarians in the first nine months of 2010. No exclusion criteria were used other than age and residence.

To encourage participation in the study, its design and aims were described in local newspaper articles and on radio programs. The study protocol was explained and discussed in meetings with local councillors, general practitioners (GPs) and local health authorities, and the resident population.

An introductory letter describing the survey was sent to all eligible residents, who were contacted by phone one to two weeks later to ascertain their intention to participate. Initial interviews took place between February 2002 and February 2004 for residents in the seven municipalities of the lower Olona valley (the orange area in Figure 1B), between January 2005 and January 2006 for residents in Gallarate, and between January and December 2009 for the centenarians residing in the other municipalities of the province of Varese. Men 95-99 years old residing in these latter municipalities were interviewed during the first six months of 2010.

Because of the advanced age, frailty and indisposition to move of the population investigated and to avoid potential bias in estimating dementia prevalence, we skipped the screening phase and opted for a one-phase study design trying to gather all the information for the entire study population at first home visit. When present, a proxy informant (usually a family member for persons living at home) was interviewed as well. Thus, at first visit, psychologists specifically trained for the survey collected information about participants' lifestyle, habits, medical history and health status from both the subject and the proxy informant who was also asked to rate the individual's everyday cognitive ability and functional disability, behavioral disturbances, as well as the stress associated with caregiving and the economic burden associated with the elderly person's health conditions. When the elderly person was not in physical or mental condition to answer the questionnaire, all the information was gathered from the proxy informant. During the same visit, testable participants were also administered a multidomain cognitive test battery.

Since dementia has been associated with shorter survival and the probability of dying at the oldest ages is very high, information on all aspects of the resident elderly who died after the prevalence day but before interview was collected from a proxy using the full questionnaire (except, of course, for the cognitive battery) and, for individuals in nursing homes, also from the institution's records. All aspects investigated refer to the time preceding death; modifications which arose as a consequence of a possible critical phase preceding death were not taken into consideration. In order to minimize attrition bias, the same was done at follow-ups for participants who died between two successive visits but at least six months after the last interview.

All participants were asked by the psychologists to agree to blood and urine tests. Samples were taken in a subsequent visit from consenting participants by a trained registered nurse or doctor at home or in the nursing home for institutionalized individuals.

To assist in the differential diagnosis of dementias, individuals with a preliminary diagnosis of dementia or suspected dementia were then asked to undergo a physical and complete neurological examination, if not already done, in their place of residence by skilled neurologists with broad experience in the diagnosis of dementias and specifically trained for the study. When needed but still not available, the neurologist proposed that the subject undergo a brain imaging study. The same schedule was repeated at each follow-up.

### Comprehensive questionnaire

The standardized questionnaire administered at home by the trained psychologists included information on demographic characteristics, educational and occupational history, socioeconomic status, social relationships, hobbies, leisure activities and interests, nutritional lifestyle, habits (smoking, alcohol, coffee and tea consumption, physical activity and exercise), exposure to environmental risk factors, family medical history (with particular emphasis on central nervous system diseases), past and present medical history, previous use of certain drug classes (anti-inflammatory agents, statins, H2 antagonists, estrogens, and "memory enhancers"), current drug use (with inspection of the drug packages), use of health services over the past year (hospital admission, emergency room, medical and instrumental investigations), assistance and supervision needs, invalidity and mobility allowance. Any medical record presented by the subject was transcribed in the questionnaire. Reported medical histories were compared and substantiated with, when available, the information gathered from GPs or geriatricians for the institutionalized elderly and from the clinical records of the three Alzheimer's disease Evaluation Units (AEUs are clinical centers qualified for the diagnosis and free-of-charge pharmacological treatment of Alzheimer's disease) located in the surveyed area. All medical information available is reviewed in the coordinating center.

Interview lasted two hours (SD 41 minutes) on average: about two hours and 20 minutes when the neuropsychological battery could be administered and one and a half hours when it could not. The trustworthiness of the interview (i.e. the cooperativeness, consistency, confidence, etc. of the participants in reporting the information) was rated by the psychologist on a 5-point scale: "very good'", "good", "sufficient", "insufficient", and "difficult to evaluate". At baseline, interviews were rated as "good" or "very good" in 80.0% of the cases, while only 0.8% were deemed "insufficient".

The reliability of the questionnaire had previously been investigated, showing high agreement: Cohen's k was between 0.84 and 0.93 [9,10]. To control for possible bias in reporting drug use, data on cholinesterase inhibitor use from the present study were compared with those from National Health Service drug prescriptions from the same area and period of the prevalence study: National Health Service data gave almost identical results to those from the present survey [11].

### Measurements

All tests and scales used are widely employed measures with good validity and reproducibility. They were administered and scored by the specifically trained psychologists following standardized criteria detailed in the instruction manual. All scores are centrally re-assessed.

#### Cognitive performance and competence

The Mini-Mental State Examination (MMSE) is a brief screening instrument designed to assess global cognitive performance [12]. The Italian version used here [13] was standardized in a cognitively normal elderly Italian population [14] and shows high test-retest reliability (n = 318, r = 0.89) [15]. In subjects with severe dementia but still testable, the Severe MMSE [16] was administered. Elderly persons with a very low educational level, serious visual deficits, or manually impaired were administered the Blessed Information Memory Concentration test (BIMC) [17,18], a brief mental status test similar to the MMSE but with no visual or manual tasks and that can be administered also to the illiterate. BIMC as well has excellent test-retest reliability as assessed in a previous study (*r *= 0.90, n = 765) [authors' unpublished data]. A set of neuropsychological tests assessing several aspects of cognition was also administered to the testable elderly: Verbal Fluency ("Animal Category") to evaluate verbal production, semantic memory, and language; the Modified Boston Naming Test (15-items) to measure visual naming skills; the Word List Memory task to assess the ability to remember newly learned information (a list of ten words); the Word List Recall to evaluate delayed recall and the Word List Recognition for delayed recognition of the ten words previously presented in the Word List Memory task; Constructional Praxis to measure visuospatial and constructional abilities and Recall of Constructional Praxis to assess visual memory. All these tests are from the CERAD battery [19] and have been previously standardized in a cognitively normal elderly Italian population [14]. Furthermore, the Clock-drawing test was administered following the CERAD protocol to explore visuo-spatial, constructional, and executive functions; the Prose Memory test to measure the immediate and delayed recall of a short story [20,21]; the Visual Search on Matrices of Digits (score range: 0-60) to test selective and sustained attention [20]. The Trail Making Test (Part A and Part B) [22,23], initially included to examine visual attention, psychomotor speed and cognitive flexibility, was then eliminated because of general poor performance and replaced with the Stroop Colour-Word test assessing selective attention and cognitive flexibility [24]. The shortened version of the Stroop test has been studied in an Italian population [25]. Judgement and problem solving ability was evaluated using Clinical Dementia Rating (CDR) interview worksheets for patient and informant [26,27].

Daily-life cognitive competence was assessed with the Social Interactions section of the Spontaneous Behavior Interview rating scale (SBI-SI) and the Informant Questionnaire on Cognitive Decline in the Elderly (IQCODE). The SBI is a multidimensional interview with a proxy informant used to measure the whole spectrum of dementia symptoms in the everyday environment and consists of three subscales assessing basic activities of daily living (bADL), social interactions (SI), and behavioral disturbances (BD). Validity and reliability of the scale and its sections have been previously investigated in a very large number of subjects [28,29]. Inter-rater and test-retest reliabilities were excellent: r = 0.96 and r = 0.97 (n = 310), respectively. The IQCODE rates changes in everyday cognitive function based on informants' reports. Mean global score ranges between 1 (much better) and 5 (much worse) [30,31]. When used retrospectively (on average 22 months after death), the IQCODE was a valid instrument to identify cognitive impairment in subjects with neuropathologically defined Alzheimer's disease [32].

The MMSE showed a very high correlation coefficient with both SBI-SI (*r *= 0.85, *p *< 0.0001) and BIMC (*r *= 0.90, *p *< 0.0001). Thus, for individuals already deceased or who could not be tested because of serious sensory deficits, acute medical condition, or refusal, a MMSE score was calculated (converted-MMSE) following a method previously described [33,34]: the score on BIMC or SBI-SI was converted to MMSE score applying the formulas provided by regression analyses (MMSE = 2.78 + [0.80 × BIMC] and MMSE = 28.10 - [0.87 × SBI-SI]). A converted-MMSE (c-MMSE) score was calculated also for individuals with advanced cognitive deterioration who were administered only the Severe MMSE.

#### Functional disability

The bADL section of the SBI assesses five domains of basic self-care ADL: dressing, eating, walking, bathing and continence. Scores range between 0 and 30, the lowest indicating no degree of dependence [28,29]. The Instrumental Activities of Daily Living scale (IADL) investigates more complex daily tasks such as the ability to use the telephone, prepare meals, handle finances, etc. [35]. The scale covers eight activities for women and five for men. Raw scores were converted to a new score indicating the percentage of dependence: this new score ranges between 0 and 100% for both men and women with the lowest score indicating no degree of dependence. IADL and SBI-bADL are instruments with very high inter-rater and test-retest reliability [28,36] and have been widely used in epidemiological and clinical trial studies.

#### Behavioral disturbances

Nine common behavioral disturbances in dementia were investigated by trained psychologists with the BD section of SBI (SBI-BD): motor agitation, psychic agitation, episodes of violence against things or people, getting lost, episodes of nocturnal confusion, eating disorders, hallucinations, delusions, and sexual disinhibition [28,29]. Apathy was evaluated in a different section of the SBI, while the complex and variegated symptomatology of depression was assessed with *ad hoc *instruments. SBI-BD assesses the presence and frequency of behavioral disturbances during the previous month on a scale from 0 (absent) to 3 (always present), where each degree of frequency is clearly defined. The total BD section score ranges from 0 to 27. Agreement was high on items similarly investigated by the Neuropsychiatric Inventory (NPI) and SBI-BD such as delusions and hallucinations: respectively 0.90 and 0.94 for the frequency of the behavior and 0.94 and 0.97 for the presence of the behavior (n = 741 Alzheimer's disease patients, authors' unpublished data).

#### Mood

The Italian version of the Geriatric Depression Scale (GDS) was devised to rate depression in the elderly [37,38]. GDS-10 is a ten-item version highly correlated with the original and showing good sensitivity and specificity for significant depressive symptomatology with a cut-off of 3/4 [39]. Scores range between 0 and 10 points, with 0 indicating absence of symptoms. When cognitive impairment was apparent, the Cornell Scale for Depression in Dementia (CSDD) was administered to subjects and/or informants. Scores range between 0 and 38, higher scores indicating more severe depressive symptomatology [40].

### Training and quality control

Psychologists were recruited locally and followed a training course on each aspect of the research until they were proficient in the standardized interview procedures. All questionnaires completed were reviewed and discussed by the study coordinators with each psychologist in weekly meetings at the beginning of the study and then monthly. The main common problems or field difficulties were discussed at monthly conferences. Even though test-retest and inter-rater reliabilities of most instruments were already known before the study started, agreement between interviewers was evaluated at one of the study follow-ups. During the first months of the survey the feasibility of the questionnaire was tested and procedure standardization was optimized. On the basis of this pilot study, a few minor revisions of practical aspects of the interview were introduced. Other small changes or supplemental material were introduced over the course of time.

### Diagnosis

The diagnosis of dementia was made according to the criteria of the *Diagnostic and Statistical Manual of Mental Disorders*, fourth edition (criteria A, B, E, and F for Dementia of the Alzheimer's Type) [41]. At the end of each interview, study psychologists described and evaluated the cognitive status of the elderly person. Subsequently, each study evaluator (a neurologist, a neuropsychologist, and a psychologist) reviewed all the information collected and, independently of the other evaluators, reached a preliminary diagnosis of dementia syndrome according to DSM IV criteria. The degree of confidence was rated on a two-plus-two decision scale: "dementia" and "probable dementia" on one side, "probable no dementia" and "no cognitive impairment" on the other. Degree of confidence reflects both the evaluators' subjective grading of the individual's cognitive functioning and the completeness and trustworthiness of the information gathered for each subject. In the case of disagreement, the experts reviewed all the data, discussed initial diagnoses, and reached a final consensus. In case of disagreement on the degree of confidence, the most prudent rating ("probable") was assigned. Diagnoses of dementia made at a certain visit but not confirmed at subsequent ones were reclassified as "non dementia" at that visit. The global severity of dementia was staged in accordance with the Clinical Dementia Rating (CDR) scale [26,27].

Based on all the information available including clinical records and previous diagnosis present, the research neurologists made an initial clinical diagnosis of the dementia type for each subject they examined according to DSM IV and other accepted diagnostic criteria. All data will be reviewed and the preliminary diagnoses discussed by a panel of experts which will reach a final diagnosis of dementia type.

Study procedures were in accordance with the principles outlined in the Declaration of Helsinki of 1964 and following amendments. The study protocol was submitted to and approved by the Local Research Ethics Committee (Azienda Sanitaria Locale of Varese Province). Psychologists and nurses or doctors obtained separate written informed consents from all participants for data and blood sample collections.

### Laboratory methods

Fasting blood samples were collected by venipuncture from consenting participants in a sitting position. Blood and urine examination included routine investigations as well as laboratory tests aimed at identifying potentially reversible dementias and aggravating factors (complete blood count, glucose, creatinine, total protein, protein electrophoresis, AST, ALT, total cholesterol, HDL cholesterol, triglycerides, sodium, potassium, calcium, vitamin B_12_, folate, TSH, FT3, FT4, TPHA, and urine test). Laboratory analyses were all done by Laboratorio Milano. All blood donors consented to the storage of a blood sample for studies of genetic epidemiology.

### Statistical analysis

To describe the baseline status of the study sample we tabulated the percentages (for nominal variables) and means and standard deviations (for numerical variables) of socioeconomic characteristics, life style habits, activities and clinical features of the population. Cognitive, functional, mood and behavior profiles were described using both means and standard deviations and medians and upper and lower quartiles so as to give a comprehensive view of the distribution of the variables, particularly for the ones that were not normally distributed. All characteristics were tabulated in the overall sample and by quinquennia. Trend across (ordered) age groups was studied using a test developed by Cuzick [42].

Characteristics derived from interview of the proxy informants were compared between subjects alive at the time of the study visit and subjects deceased after prevalence day but prior to the study visit by means of chi-squared (for nominal variables) or Student's t-test (for numerical variables).

The correlation between cognitive performance and daily life cognitive competence was calculated by means of Pearson's r linear coefficient.

Elderly subjects with and without MMSE were compared on a number of characteristics retrieved from the interviews with proxy informants by means of chi-squared (for nominal variables) or Student's t-test (for numerical variables). Comparisons were repeated to adjust for age, sex and education using ANOVA.

Student's t-test and Pearson's linear correlation coefficient results were cross-checked with the non parametric Mann-Whitney test and Spearman's rank correlation coefficient, respectively, finding very similar results (and no change from significance to non significance or viceversa).

Analyses were done using JMP v9.0.1 (Sas Institute Inc., Cary, NC) and Stata/IC v11.1 (StataCorp, College Station, TX).

## Results

### Study population

**Figure 2:**
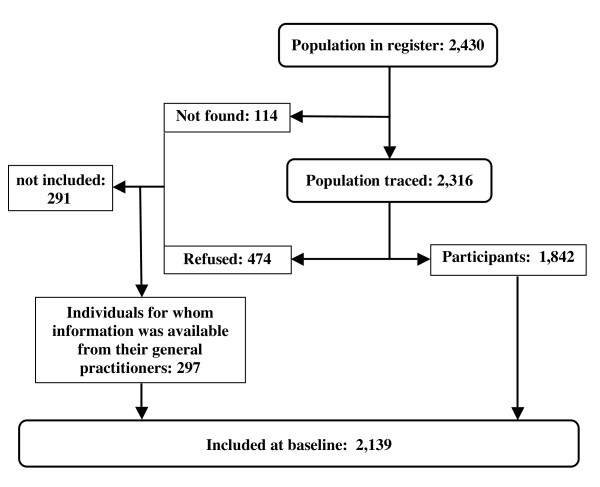
**Flow chart of the Monzino 80-plus Study**.

Figure 2 shows the flow diagram of the study. Of the 2,430 individuals 80 years or older registered in the municipalities, 114 could not be traced. Among the 2,316 traced, 474 refused and 1,842 agreed to participate. A parallel study with the GPs of the same population provided information, in an anonymous form (individuals were identified only by age at prevalence day and sex), on the medical history of a further 297 elderly persons among those who refused or could not be found. Thus 2,139 individuals (88.0% of the registered population, 92.4% of the traced population) were included in the main analyses. Since interviews of centenarians and those of men 95-99 years old have just been concluded, data on these individuals are not yet available for analyses and are thus not reported in Figure 2 and the Tables. The response rate in these age groups was very high as well (95%).

Demographic characteristics of the entire population and of participants and non-participants are reported in Table 1. Percentage of women and mean age were very similar between the original and the investigated populations (respectively: 73.9% and 74.1%, 87.2 and 87.3), but a little lower in the small group of elderly for whom no information was available (72.2% and 86.3). The percentage of elderly persons who died in the year after the interview or, for those without interview, after the prevalence day, was similar for the 2,430 residents (12.8%), 1,842 participants (12.8%) and 588 elderly persons without interview (12.9%).

**Table 1:** Baseline demographic characteristics of the original population, the population included in the analyses, the participants, and the group of elderly without information

	Residents	Individuals with information	Participants	Individuals without information
All, n	2,430	2,139	1,842	291
Men, n (%)	634 (26.1%)	553 (25.9%)	469 (25.5%)	81 (27.8%)
Women, n (%)	1,796 (73.9%)	1,586 (74.1%)	1,373 (74.5%)	210 (72.2%)
Mean age of men at interview (SD), years		86.9 (4.6) ^1^	87.2 (4.5)	
Mean age of women at interview (SD), years		88.3 (4.8) ^1^	88.7 (4.7)	
Mean age at interview, all (SD), years		88.0 (4.8) ^1^	88.4 (4.7)	
Mean age at prevalence day, all (SD), years	87.2 (4.9)	87.3 (5.0)	87.6 (4.9)	86.3 (4.9) ^2^
Died in the year following interview, ^3 ^%	12.8%	n.a. ^4^	12.8%	n.a. ^4^

^1 ^At prevalence day for the individuals without interview; ^2 ^Approximate estimates of the SD; ^3 ^After prevalence day for those without interview; ^4 ^The death rate was 12.9% in the 588 individuals without interview (297 with and 291 without information).

### Socioeconomic characteristics, life style habits and activities

Table 2 shows the socioeconomic characteristics of participants and informants. As expected in an Italian population of this age, the mean level of education was low: about five years, corresponding to the completion of elementary school. The education of the informants was limited, but sensibly higher: it averaged more than eight years, corresponding to the completion of lower secondary school. The majority of the population (77%) had practised a manual occupation before retirement. More than one third were living alone at least until age 95. The percentage of the oldest olds living in institutions rose steeply with age, from 3.5% at age 80-84 to more than a quarter over 94 years. About one in ten elderly persons rated her/his economic condition as "insufficient". For more than three fourths of the community-dwelling elderly the informant was a family member, either a spouse (12.4%) or a child (64.4%), and in almost 20% another relative (Table 2). This explains the relatively high mean age of the informants (more than 60 years).

**Table 2:** Baseline socioeconomic characteristics of participants and informants by age group

	80-84	85-89	90-94	95+	All
n	542	581	569	150	1,842
Mean education (SD), years	5.5 (2.6)	4.9 (2.0)	5.1 (2.9)	4.7 (2.4)	5.1 (2.5)
Main occupation ^1^, %					
manual workers	76.3	81.4	69.9	77.9	76.0
crafts and trades	11.3	10.8	16.3	11.0	12.7
professionals and clerks	11.7	6.6	11.9	8.3	9.9
Living alone,%	37.5	38.0	35.3	19.8	35.8
Living in institutions, %	3.5	10.9	16.3	26.0	11.6
Economic condition, insufficient, %	8.4	14.4	7.3	13.3	10.4
Home ownership, %	94.4	91.9	92.2	93.9	92.9
Principal informant, child or spouse, %	80.3	76.1	75.2	75.4	76.8
Informants' mean age (SD), years	61.4 (13.7)	59.6 (11.1)	61.6 (9.6)	64.4 (9.8)	61.1 (11.3)
Informants' mean education (SD), years	8.1 (3.7)	8.5 (3.9)	8.9 (3.9)	8.7 (3.8)	8.5 (3.9)

^1 ^Organizing the major groups of the International Standard Classification of Occupations (ISCO-08) in three classes.

Life style habits and activities are reported in Table 3. Not only were there few current smokers among the oldest old (2.6%), but the percentage of former smokers was also low (17.3%), a predictable consequence of the high proportion of women who, as customary in this age group, had never smoked (94% versus 39% among men). More than a half of the population drank on average 1.5 glasses of wine a day, with a higher prevalence among men (70% drinking on average two glasses a day) than women (47% drinking on average 1.5 glasses a day). More than 80% of this population drank coffee, a very common habit in Italy, with no appreciable sex differences. Physical activity, walking, hobbies, and social activities all decreased with age but were more common among men than women.

**Table 3:** Life style habits and activities of participants (n = 1,842) by age group at baseline

	80-84	85-89	90-94	95+	All	Men	Women
Current smoker, %	3.7	2.9	1.6	0.7	2.6	6.2	1.3
Former smoker, %	26.3	15.6	12.3	10.7	17.3	54.4	4.6
Current alcohol consumption, %	58.5	52.7	51.2	39.3	52.8	69.9	47.0
Former alcohol consumption, %	12.2	16.8	16.0	22.0	15.6	17.7	14.9
Mean current daily alcohol, cl (SD) ^1^	17.3 (12.3)	18.2 (15.5)	14.9 (10.8)	15.4 (12.5)	16.7 (13.0)	23.3 (15.7)	13.4 (9.8)
Mean former daily alcohol, cl (SD) ^1^	25.9 (34.3)	18.8 (21.3)	14.2 (12.1)	21.5 (32.0)	19.2 (24.4)	28.8 (35.9)	15.3 (16.2)
Coffee drinking, %	81.6	83.2	78.7	78.8	81.0	82.1	80.6
Physical activity, %	53.8	35.5	25.0	16.0	36.0	49.3	31.5
Walking, %	87.4	77.1	63.3	51.0	73.7	79.7	71.7
Hobbies, %	79.2	67.5	53.4	39.2	64.3	74.4	60.8
Social activity, %	48.2	37.0	20.8	12.6	33.3	35.7	32.5

^1 ^About 12 cl in one glass of wine.

### Clinical characteristics

Table 4 shows the main clinical features of the population by age group. Except for the prevalence of serious hearing and visual impairments, which increased with age, the frequency of most diseases peaked at earlier ages. As expected, osteoarthrosis and cardiovascular diseases were among the most prevalent clinical conditions. More than one person in five reported a fall and almost 4% a bone fracture in the year preceding the interview. During the same period, almost one third of the elderly population was admitted to a hospital. Based on the initial diagnoses, the preliminary estimate of over-eighties affected by dementia is rather high accounting for about one third of the present study population (32%, 35% in women and 24% in men), with prevalence increasing with advancing age (16% at 80-84 years, 34% at 85-89, 43% at 90-94, and 57% over 94).

**Table 4:** Main clinical features of the population with available information (n = 2,139) by age group at baseline

	80-84	85-89	90-94	95+	All
n	691	653	632	163	2139
Hypertension, %	58.2	57.7	56.0	49.7	56.7
Diabetes, %	15.3	12.3	13.0	9.3	13.2
Myocardial infarction, %	8.8	7.0	7.5	4.6	7.5
Heart failure, %	22.3	27.8	30.6	29.4	27.0
Chronic obstructive pulmonary disease, %	14.7	19.8	19.2	15.5	17.7
Stroke, %	10.5	14.5	13.1	15.5	12.9
Serious hearing impairment, %	1.9	6.4	9.0	14.7	6.3
Serious visual impairment, %	3.3	5.8	8.5	14.7	6.5
Osteoarthrosis, %	64.9	63.6	70.7	68.3	66.5
Hospital admission in previous year, %	35.4	32.1	30.4	26.2	32.1
Drug use, %	85.2	90.6	91.4	90.5	89.1
Mean number of drugs among users, (SD)	3.1 (1.7)	3.5 (2.0)	3.5 (2.0)	3.3 (1.9)	3.4 (1.9)
Cholinesterase inhibitors, % users	1.6	1.4	0.5	0.0	1.1
Nootropics, % users	0.3	1.5	1.9	1.3	1.2

### Cognitive, functional, mood, and behavioral assessments

Cognitive performance, cognitive competence and functional abilities decreased steadily with age (Table 5). Instead, depressive symptoms and behavioral disturbances did not show substantial variations with age. Global cognitive performance (MMSE or converted-MMSE) was highly correlated with daily-life cognitive competence ratings: Pearson's linear correlation coefficient - 0.82 and - 0.85 with IQCODE; - 0.85 and - 0.94 with SBI-SI. The accuracy of the formulas provided by regression analyses to estimate a c-MMSE score in individuals who did not perform the test was empirically tested in the sub-population of oldest old for whom the actual scores of MMSE and BIMC or of MMSE and SBI-SI were available. In the 1,268 individuals with both baseline MMSE and SBI-SI scores available, the mean MMSE score was 22.44 (SD 7.37) and the mean estimated c-MMSE score was 22.90 (SD 6.45). In the 161 individuals with both MMSE and BIMC scores available, the mean MMSE score was 17.81 (SD 7.10) and the mean estimated c-MMSE score was 17.99 (SD 6.07).

**Table 5:** Mean (SD) and median (25th-75th percentiles) baseline cognitive, functional, mood, and problem behavior scores by age group

	n	80-84	85-89	90-94	95+	All
Mini-Mental State Examination (MMSE) or converted-MMSE	1842	24.6 (6.5)	21.3 (7.4)	19.7 (7.9)	16.3 (8.1)	21.4 (7.8)
		27 (24-28)	24 (17-27)	22 (14-26)	17 (10-24)	24 (17-27)
Mini-Mental State Examination	1268	25.5 (5.7)	22.0 (7.0)	20.6 (7.7)	16.8 (8.6)	22.4 (7.4)
		27 (25-29)	24 (19-27)	23 (16-26)	18 (11-24)	25 (20-28)
Blessed-Information Memory Concentration test ^1^	37	15.8 (4.3)	12.7 (6.7)	10.4 (5.3)	10.0 (5.7)	12.4 (6.2)
		16 (12-20)	10 (8-17)	10 (5-13)	10 (6-14)	10 (8-16)
Severe Mini-Mental State Examination ^1^	11	-	21.4 (7.6)	11.2 (4.0)	-	15.8 (7.7)
		-	24 (14-28)	12 (8-15)	-	14 (9-24)
Informant Questionnaire on Cognitive Decline in the Elderly	1664	3.5 (0.6)	3.8 (0.7)	4.0 (0.8)	4.3 (0.7)	3.8 (0.8)
		3.2 (3-3.8)	3.6 (3.2-4.6)	4.0 (3.3-5)	4.4 (3.5-5)	3.6 (3.2-4.6)
Spontaneous Behavior Interview-Social Interaction	1829	4.7 (7.7)	8.1 (9.0)	10.2 (9.6)	13.3 (10.2)	8.1 (9.3)
		1 (0-5)	4 (1-13)	7 (2-16)	12 (5-20)	4 (1-13)
Spontaneous Behavior Interview-basic activities of daily living	1836	3.9 (7.3)	6.9 (8.5)	9.8 (9.6)	14.3 (9.7)	7.5 (9.1)
		0 (0-4)	4 (0-12)	6 (1-18)	15 (6-22)	3 (0-13)
Instrumental Activities of Daily Living, % disability	1829	29.6 (33.2)	49.7 (35.0)	60.7 (35.1)	77.9 (28.4)	49.5 (37.1)
		18 (0-42)	42 (20-88)	66 (27-97)	94 (63-100)	42 (17-93)
Geriatric Depression Scale-10 items ^2^	1406	2.0 (2.5)	2.9 (2.7)	2.9 (2.7)	2.8 (2.6)	2.6 (2.7)
		1 (0-3)	2 (1-4)	2 (1-5)	2 (1-5)	2 (0-4)
Spontaneous Behavior Interview-Behavioral Problems	1822	0.7 (2.0)	1.4 (2.7)	1.1 (2.5)	1.0 (2.0)	1.1 (2.4)
		0 (0-0)	0 (0-2)	0 (0-1)	0 (0-1)	0 (0-1)

^1 ^Individuals without MMSE; ^2 ^Individuals with a possible or manifest cognitive impairment were administered the Cornell Scale for Depression in Dementia (n = 716).

### Comparison of elderly with and without MMSE

Elderly persons alive at baseline without MMSE had significantly worse cognitive competence and functional ability, and reported higher prevalences of depressive symptoms and problem behaviors than elderly with MMSE (all p values < 0.0001) (Table 6). The percentages of elderly persons without MMSE rose rather evenly with age (p < 0.0001) (Table 6).

**Table 6:** Baseline characteristics of living individuals with MMSE or c-MMSE scores

	Individuals with MMSE	Individuals with c-MMSE	**p-value **^**1**^
Age group, years, % (n)			
80-84	77.0% (418)	23.0% (125)	< 0.0001
85-89	70.5% (409)	29.5% (171)	
90-94	62.4% (355)	37.6% (214)	
95+	57.3% (86)	42.7% (64)	

All, % (n = 1,842)	68.8% (1,268)	31.2% (574)	
with BIMC or SMMSE but no MMSE, n		48	
Education, mean (SD)	5.3 (2.6)	4.7 (2.4)	< 0.0001
Living in institution, % (n)	9.9% (126)	15.3% (88)	0.0007
MMSE or c-MMSE, mean (SD)	22.4 (7.4)	18.7 (8.0)	< 0.0001^2^
IQCODE, mean (SD)	3.7 (0.7)	4.1 (0.8)	< 0.0001^2^
Spontaneous Behavior Interview-SI, mean (SD)	6.5 (8.2)	12.0 (10.4)	< 0.0001^2^
Spontaneous Behavior Interview-bADL, mean (SD)	5.5 (7.8)	11.9 (10.4)	< 0.0001^2^
IADL, mean % disability (SD)	42.0 (35.6)	66.0 (35.1)	< 0.0001^2^
Geriatric Depression Scale-10, mean (SD)	2.3 (2.4)	4.0 (3.2)	< 0.0001^2^
Spontaneous Behavior Interview-BP, mean (SD)	0.9 (2.1)	1.8 (3.1)	< 0.0001^2^

BIMC: Blessed Information Memory Concentration test; SMMSE: severe Mini-Mental State Examination; c-MMSE: converted-MMSE; IQCODE: Informant Questionnaire on Cognitive Decline in the Elderly; SI: Social Interaction; bADL: basic Activities of Daily Living; IADL: Instrumental Activities of Daily Living; BP: Behavioral Problems.

^1^Student's t-test for numerical variables, chi-squared for nominal variables. ^2^Both univariate and adjusted for age, sex and education.

### Comparison of alive elderly and those who died

Table 7 shows the main baseline characteristics of living elderly persons and of those who died between the prevalence day and the first visit for whom information was available. On average, informants were interviewed about ten months after the individual's death. In comparison with those living, elderly persons who were already dead at the time of the first visit were older, had twice the rate of institutionalization, lower cognitive competence, and significantly higher instrumental and basic functional disability.

**Table 7:** Baseline characteristics of the elderly alive at first visit and those who died between the prevalence day and first visit

	Alive	Dead	**p value **^**1**^
All (1,842), n (%)	1688 (91.6%)	154 (8.4%)	
Men, n (%)	427 (91.0%)	42 (9.0%)	0.5899
Women, n (%)	1261 (91.8%)	112 (8.2%)	
Age, mean (SD)	88.2 (4.6)	89.5 (5.2)	0.0036
Age group, years, (%)			0.0059
80-84	93.5	6.5	
85-89	92.9	7.1	
90-94	90.0	10.0	
95+	86.0	14.0	
Education, years, mean (SD)	5.1 (2.6)	5.0 (2.1)	0.3019
Living in institutions, %	10.7%	21.4%	<0.0001
Mini-Mental State Examination (MMSE) or c-MMSE, mean (SD)	21.5 (7.7)	19.9 (8.4)	0.0290
Spontaneous Behavior Interview (SBI)-Social Interactions, mean (SD)	7.9 (9.2)	10.4 (10.8)	0.0064
Informant Questionnaire on Cognitive Decline in the Elderly, mean (SD)	3.8 (0.7)	4.0 (0.8)	0.0029
Instrumental Activities of Daily Living, mean % disability	48.1 (37.0)	64.9 (35.4)	<0.0001
SBI-basic Activities of Daily Living, mean (SD)	7.1 (8.9)	12.0 (10.3)	<0.0001
SBI-Behavioral Problems, mean (SD)	1.1 (2.4)	1.0 (2.5)	0.6981

c-MMSE: converted-MMSE; SBI: Spontaneous Behavior Interview. ^1 ^Student's t-test for numerical variables, chi-squared for nominal variables.

## Discussion

Until recently, relatively few data have been available on the epidemiology of dementia in the oldest old [5], despite its being the fastest growing age group and the most cognitively impaired one.

Most of the numerous population-based studies on cognitive impairment and dementia have included a broad age span of elderly persons (commonly 65 years or older) with the consequence that the number of oldest old investigated has usually been too small to accurately estimate the prevalence and incidence of dementia in this segment of the elderly population. Even when the number of oldest old included was large enough [43-53], extreme ages were often poorly represented. To overcome this limitation and more reliably investigate the relationship between age and dementia in the advanced ages, a few studies have focused on the oldest old. All available inhabitants aged 85 years or older (n = 891; 95+ years = 34) were included in the "Leiden 85 plus study" [54]. A random sample of 358 citizens aged 85 years and over (90+ years = 91) was assessed in the Munich study [55]. Prevalence and severity of dementia was investigated in two birth cohorts of 494 85-year-old and 338 95-year-old persons born in Göteborg [56,57]. The Vantaa 85+ Study included all available persons of at least 85 years of age (n = 553; 90+ years: 105 out of 399 deceased individuals) but only the overall point prevalence of dementia was reported [58]. Survivors aged 90 and older (n = 911) from the Leisure World Cohort Study, a retirement community in California, were enrolled in the 90+ Study [59]. Information on these populations of oldest old was mostly limited to some demographic characteristics while no or very few data on general health status, life style habits, functional disability, and behavioral disturbances have yet been reported.

Conducting studies in the oldest old is of course much more problematic than in the younger old. Assessing and monitoring cognitive behavior in a population with high morbidity and disability is challenging: many die, many are in critical condition or seriously ill, have severe sensory or language deficits, are easily fatigued, or do not cooperate. Moreover, feeling vulnerable and defenceless makes the very old and their relatives more hesitant to agree to participate in research studies. In consideration of this complexity, our initial choice to conduct almost all of the aspects of the research at the elderly's place of residence proved successful. This was confirmed by the widespread unwillingness of the elderly with dementia to carry out a brain imaging study to assist in the differential diagnosis of dementia type.

Relying on a large array of diverse tools to assess the multifaceted manifestations of dementia has also proved successful. Informant-based assessment instruments of cognitive behavior such as SBI-SI and IQCODE not only showed less attrition than measures that required testing the individual [60]; because of their very high correlation with measures of cognitive performance, it was also possible to evaluate individuals who could not or refused to be tested and thus compare the cognitive functioning and change in this group to that of the testable elderly.

### Habits, health status and the risk of cognitive decline

Despite the poor health of many elderly persons, more than one third of this population of oldest old were living alone, though in several cases in close contact with children and relatives. The frequency of most diseases did not continue to rise at the extreme ages, suggesting that those who reach advanced years (for the most part women) might be a select and particular group. However, cognitive performance and competence as well as instrumental and basic activities of daily living all steadily decline with age.

Lifestyle habits like smoking and, to a lesser degree, alcohol and coffee consumption also tended to decrease with age. Though a small percentage of oldest men were still smoking, more than half were former smokers, while only a small fraction of the oldest women had ever smoked. Behaviors deemed to be protective like physical and social activities and hobbies also tended to decrease with age. It is of note that in the face of the loss of cognitive and functional abilities, the prevalence of putative risk factors for cognition like diabetes declines with age. This seems to suggest that other age-related factors would intervene, negatively affecting the brain and that different pathogenetic mechanisms could be involved in dementia onset depending on age.

### Cognitive performance in the oldest old

In the present study population performance on the MMSE steadily decreased with increasing age (Table 5). Using the same standardized version and administration and scoring procedures of the MMSE in a population of 1,680 cognitively normal overeighties from four Italian communities (mean education: 6.2 years), we found that the mean MMSE score declined slowly with increasing age: 26.9 at age 80-84, 25.7 at age 85-89, 25.2 at age 90-94, and 24.4 at age 95 and over (unpublished data). Difference in mean MMSE score between this cognitively normal population and the present study general population (Table 5) progressively increased with increasing age (1.4 at age 80-84, 3.7 at age 85-89, 4.6 at age 90-94, and 7.6 at 95 and over) strongly suggesting that in the general population the proportion of oldest old with cognitive impairment rises with age. Mean MMSE score in the present study population was consistently lower also compared to that of other populations with a higher educational level. Without considering possible item variations and different administration and scoring procedures of MMSE in the diverse studies, the mean MMSE score of the present study population was 22.4 (n = 1,268; 0-8 years of education: 93.1%) versus 24.6 in the 80 years or older individuals of five US community populations (n = 951; 0-8 years of education: 56.9%) [61] and 23.9 in the 80 years or older individuals of the Kungsholmen Project (n = 1,067; 0-7 years of education: 26.4%) [62]. In a sample of 435 adults aged 90 or older enrolled in the 90+ Study, a well-educated, upper middle class cohort, the mean MMSE score was 22.2 (0-12 years of education: 26.7%) [63] versus 19.9 in the 90 and older individuals of the present study (n = 441, 0-12 years of education: 95.7%). The two Göteborg cohorts had a higher proportion of elderly with a low educational level (0-6 years: 75% in the 85-year-old cohort and 67% in the 95-year-old cohort) and their mean MMSE scores (~23.5 in the 85-year-old cohort and ~18.1 in the 95-year-old cohort) were similar to those of the individuals of the same age in the present study (85-year-old: 23.4; 95-year-old: 18.5) [56,57]. These results suggest that the comparison of prevalence and incidence rates of dementia between populations with quite different educational levels could represent an interesting model to investigate the actual and still controversial role of formal education in cognitive decline and in the development of dementia and Alzheimer's disease.

### Failing to consider the deceased or untested individuals results in bias

The results reported in Tables 6 and 7 have noteworthy implications. The lack of MMSE does not appear to be a random, innocent occurrence. Subjects alive and without MMSE are clearly older, more often institutionalized, and have much worse cognitive, functional, and behavioral profiles than those with MMSE. Hence the importance of also measuring observable behaviors in the everyday environment to gather more complete and reliable information in a field survey on the very old. Collateral sources, usually the spouse or an adult child, were shown to be accurate in reporting the cognitive abilities of subjects even in a very mild stage of dementia [64,65]. Moreover, informant-based instruments help establish the relevance of current deficits in cognitive tests to everyday functioning and assess change from the pre-morbid level or from a previous disease stage. This longitudinal approach proved to be a valid aid in the diagnostic process.

Shorter survival is associated with a higher level of cognitive impairment [66]. Dementia shortens life expectancy [67], even in the oldest old [68-71]. This differential mortality between demented and non-demented elderly can influence prevalence and incidence estimates of dementia [72,73] and cognitive impairment [74], particularly among the oldest old. What emerges from comparisons of the demographic and clinical characteristics of individuals still alive versus those who had died at the time of the first visit (Table 7) is that the failure to include information on deceased elderly would increasingly underestimate with age the prevalence of cognitive and functional disability in the elderly population. Retrospective interviews (on average two years after death) were shown to be sensitive instruments to detect the antemortem presence of dementia and were strongly associated with late-life MMSE scores taken close to death [32,75,76]. In the attempt to limit the attrition bias due to the expected high mortality rate and differential survival rates, a great effort was made at follow-ups to gather complete information also on participants who had died between two subsequent visits.

## Conclusions

The Monzino-80 Plus is one of the largest prospective population-based studies specifically aimed at thoroughly investigating cognitive decline and dementia in the very old. The large number of individuals investigated also at extreme ages, the high response rate, one-phase design, extensive baseline information collected with standardized and reliable instruments, and close attention to keeping the population representative over time should provide more accurate estimates of the occurrence of dementia in the oldest ages and contribute significantly to understanding the complex, heterogeneous basis of cognitive decline and dementia. Failure to take deceased or untested individuals into consideration could affect the accuracy of the estimates of prevalence of cognitive and functional disability.

## Competing interests

The authors declare that they have no competing interests.

## Authors' contributions

UL, MG, MT conceived and designed the study. UL, MG, PT, MF, CB, AB, EG, MM, AN, LP, CF, ER, MT performed data collection and management. UL, MG, AR, GL, PT, MT analyzed and interpreted the data. UL drafted the manuscript. All authors critically reviewed the manuscript and approved the final version.

## Pre-publication history

The pre-publication history for this paper can be accessed here:

http://www.biomedcentral.com/1471-2377/11/54/prepub
